# Effects of an individualised nutritional intervention to tackle malnutrition in nursing homes: a pre-post study

**DOI:** 10.1007/s41999-021-00597-y

**Published:** 2021-12-01

**Authors:** J. Seemer, E. Kiesswetter, D. Fleckenstein-Sußmann, M. Gloning, S. Bader-Mittermaier, C. C. Sieber, B. Sixt, S. Wurm, D. Volkert

**Affiliations:** 1 Institute for Biomedicine of Aging, Friedrich-Alexander-Universität Erlangen-Nürnberg, Nuremberg, Germany; 2 Fraunhofer Institute for Process Engineering and Packaging, Freising, Germany; 3 Institute of Food Technology, Hochschule Weihenstephan-Triesdorf, Freising, Germany; 4 Department of Medicine, Kantonsspital Winterthur, Winterthur, Switzerland; 5 Institute for Community Medicine, Department of Prevention Research and Social Medicine, Universitätsmedizin Greifswald, Greifswald, Germany

**Keywords:** Malnutrition, Nursing home, Individualised, Nutritional intervention, Energy intake, Food supplements

## Abstract

**Purpose:**

Individualised interventions are recommended to tackle malnutrition in older adults, but approaches for nursing home (NH) residents are scarce. This study investigated the effects of an individualised nutritional intervention in NH residents with (risk of) malnutrition.

**Methods:**

In a pre-post study, 6 weeks (w) of usual care were followed by 6w of intervention. The intervention consisted of up to three supplement modules (sweet and savoury protein creams and protein-energy drink, single or combined) and, if required, reshaped texture-modified meals (RTMM).

**Results:**

Fifty residents completed the study (84 ± 8 years, 74% female). One-third (32%) received RTMM. Additional 258 ± 167 kcal/day and 23 ± 15 g protein/day were offered. Mean daily energy intake increased by 207 (95%CI 47–368, *p* = 0.005) kcal and protein intake by 14 (7–21, *p* < 0.001) g (w12 vs w1). Quality of life (QoL) increased in the subscale “care relationship” (+ 9 (3–15) points, *p* = 0.002, w12 vs w6). Body weight, handgrip strength, and other QoL subscales did not change.

**Conclusion:**

Our intervention improved dietary intake and one QoL subscale in NH residents with (risk of) malnutrition. As a next step, randomized controlled trials are needed to investigate the impact of individualised interventions more comprehensively.

**Supplementary Information:**

The online version contains supplementary material available at 10.1007/s41999-021-00597-y.

## Key summary points

**Aim:**

The aim of this study was to investigate the effects of an individualised nutritional intervention on dietary intake (primary outcome), body weight, handgrip strength and quality of life in nursing home residents with (risk of) malnutrition.

**Findings:**

Our individualised nutritional intervention consisting of three supplement modules (offered single or combined) and reshaped texture-modified meals (for residents with chewing and/or swallowing difficulties) improved energy and protein intake and one quality of life subscale.

**Message:**

In this pre-post intervention study (*n* = 50) the individualised nutritional supplementation, reshaped texture-modified meals and potentially increased awareness by nurses improved primary outcomes. Future research should investigate the impact of individualised interventions more comprehensively, in randomized controlled trials and in larger samples.

**Supplementary Information:**

The online version contains supplementary material available at 10.1007/s41999-021-00597-y.

## Introduction

In European nursing homes, about one in five residents is affected by malnutrition, and almost half are at risk [1]. Malnutrition is a relevant problem, also on a global level [2], leading to numerous negative consequences, e.g., functional impairment [3], reduced quality of life [4] and premature death [5]. A major risk factor for malnutrition, which is present in about one in four residents [6], are swallowing difficulties [7]. To ensure swallowing safety and facilitate chewing, texture modifications are used [7]. Pureeing or mashing of meals can however negatively affect appearance and taste [8] and consequently energy and protein intake [9]. Hence, an optimization of texture-modified meals is desirable [10].

Besides swallowing difficulties, nursing home residents are affected by a variety of risk factors, including recent hospitalisations [11], pressure ulcers [11], dementia [12], and functional impairment [12]. These risk factors can contribute to decreased energy and protein intake, increased dietary requirements and therefore, deficiencies [13]. In clinical practice, fortification of food and oral nutritional supplements (ONS) are used to improve dietary intake [14]. To adequately address nutritional problems and needs, guidelines recommend the individualisation of interventions [15].

In hospital patients at nutritional risk, individualised nutritional support increased dietary intake, improved quality of life and lowered the risk of adverse clinical outcomes [16]. Studies in nursing home residents so far examined the effects of individualised interventions mainly within multicomponent strategies (for example nutrition in combination with group exercises, oral care, or occupational therapy), but not the effects of individualised nutritional interventions alone [17–19].

Accordingly, we first developed an individualised approach to tackle malnutrition in nursing home residents by combining optically optimized reshaped texture-modified meals with three supplement modules according to individual dietary requirements [20]. The aim of the present pre-post intervention study was to examine the effects of this individualised concept on dietary intake, body weight, handgrip strength and quality of life in nursing home residents with (risk of) malnutrition.

## Materials and methods

### Study design

**Fig. 1:**
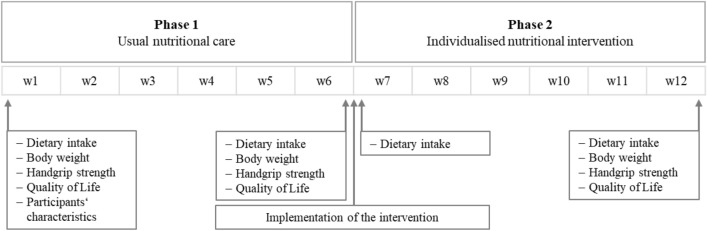
Study design and measurements. *w* week

This prospective study was conducted in two nursing homes from the same municipal provider producing meals in one central kitchen. In a pre-post design, residents received 6 weeks of usual care (Phase 1) followed by 6 weeks of individualised nutritional intervention (Phase 2) (Fig. 1). Kitchen and nursing staff were trained in the last weeks of phase 1 to implement the intervention from week 7. The trial was registered at drks.de (DRKS00017584).

### Ethical statement

The study was in accordance with the Declaration of Helsinki about ethical principles for medical research. Approval was given by the ethics committee of Friedrich-Alexander-Universität Erlangen-Nürnberg (Reference: 71_19 B). All participants or their legal representatives provided written informed consent.

### Participants

All residents living permanently in the nursing homes were screened for (risk of) malnutrition by nursing staff supported by research associates. Malnutrition was defined according to the Mini Nutritional Assessment-Short Form (MNA-SF) ≤ 7 points [21, 22]. Risk of malnutrition was identified either by MNA-SF 8–11 points and a reduced score in at least one of the following MNA-SF questions: decreased food intake (A, < 2 points), unintentional weight loss (B, < 3 points), psychological stress or acute disease (D, < 2 points) and/or low Body Mass Index (BMI, F, < 3 points) [21]; or by receiving texture-modified diet and a reduced score in one of the described MNA-SF questions.

Exclusion criteria were age < 65 years, enteral or parenteral nutrition, acute illness, terminal stage of life (according to nurses’ estimation) and BMI ≥ 30 kg/m^2^.

### Usual nutritional care

Usual nutritional care is described in detail in Supplement Fig. S1 and in previous publications [20, 23]. Briefly, residents received three main meals (breakfast, lunch and dinner) and additional snacks, delivered from the central kitchen. Breakfast and dinner were based on bread and pastries, butter, cold cuts, cheese, jam (for breakfast) and daily specials (e.g., pickled salad, fish; for dinner). Residents with chewing and/or swallowing difficulties received porridge. For dinner, an additional soup could be ordered. If desired, residents received yoghurt. For lunch, residents could choose their meals weekly out of three menu lines (one vegetarian), consisting of soup, main course and dessert. Two menu lines were offered in texture-modified form. Between main meals, snacks were offered (e.g., yoghurt, fruit and pastries). Additionally, residents could consume meals and snacks brought by family and friends. Water and juice were freely available at all times. For breakfast and during the afternoon snack, coffee and tea was provided.

If perceived necessary by nurses and/or physicians, residents received ONS or maltodextrin (stirred into meals by nurses) in addition to usual meals and/or energy enriched soups instead of usual soups.

### Individualised nutritional intervention

The intervention consists of reshaped texture-modified meals and combinations of three food supplements. It was based on the usual nutritional care concept of the nursing homes.

Residents receiving texture-modified meals during usual the care phase received optically optimized reshaped texture-modified meals during the intervention phase. These were derived from the daily menu of the nursing homes. Meal components were separately pureed, reshaped using texturizers and silicone moulds, shock-frosted and reheated [8]. One texture level was provided.

Regardless of the meal texture, three supplement modules (two protein creams and a protein-energy drink) were offered single or combined in five levels to compensate for individual energy and protein deficiencies. Energy and protein deficiencies were calculated as the difference between intake (assessed by 3-day weighed food records) and requirements at baseline. Energy requirements were estimated by multiplying calculated resting energy expenditure (based on body weight, age and sex) [24] by physical activity level (1.2 for inactive, e.g., bedridden; 1.4 for moderately active, e.g., independent walking or use of a manual wheelchair; 1.6 for very active, e.g., going out almost daily, hyperactivity in dementia) [25]. Protein requirements were calculated using 1 g protein per kg body weight [25] or 0.8 g per kg body weight in case of renal disorders [26]. Based on energy and protein deficiency levels residents were assigned to the corresponding level of supplementation (Supplement Table S1) during structured individual case discussions within the study team taking BMI, weight objective, dietary habits and expected acceptance into account. In case of any uncertainties regarding the assignment of the appropriate supplementation level, nursing staff was consulted [20]. Food supplements were offered in addition to usual nutritional care.

The protein creams were freshly produced in the kitchen in a sweet and a savoury variant (made of cream, whey protein and cinnamon, additionally powdered and vanillin sugar were added for the sweet variant and spices and maltodextrin for the savoury variant), each portion (40 g) containing 125 kcal and 10 g of whey protein, and delivered on the lunch tray of the respective participant. The protein-energy drink was specifically developed and produced for this study by Fraunhofer IVV (Freising, Germany) [20] and offered in a 250-mL ready-to-drink preparation containing 220 kcal and 22 g protein (made of whey protein, mango-fruit-preparation, yoghurt powder, sugar and vegetable oil). The protein-energy drink was labelled by research associates (day and name of participant) and delivered to the nursing wards on a weekly basis. Nurses were instructed to offer the drink at once or in several portions spread throughout the day, according to participants’ liking.

In supplementation level 1, the sweet protein cream (+ 125 kcal, + 10 g protein), in level 2, either the combination of sweet and savoury protein cream or the drink (+ 220–250 kcal, + 20–22 g protein), in level 3, the sweet protein cream and the drink (+ 345 kcal, + 32 g protein) and in level 4, both protein creams and the drink (+ 470 kcal, + 42 g protein) were offered daily to the participants. Residents with adequate intake did not receive a supplementation (level 0) (Supplement Table S1).

### Measurements

#### Participants’ characteristics

Participants’ characteristics extracted from care records included sex, age, medication, chronic diseases, swallowing disorder (diagnosed by a physician) and body height. Clinical Frailty Scale (CFS, 1–9 [27]), activities of daily living (Barthel-Index, 0–100 [28]), dementia (severe, mild, or no dementia), mobility (bed/chair bound, able to get out of bed/chair but does not go out, or goes out [21]) and eating assistance (partial/full assistance or guiding/no assistance) were assessed in personal interviews with responsible nursing staff.

#### Adverse events

Adverse events (gastrointestinal complaints, hospital stays) were documented in daily routine by nursing staff during the study and transferred from care records by research associates.

#### Outcomes

*Energy and protein intake* (primary outcome) were assessed by weighing all offered food and leftovers on 3 consecutive days in weeks 1, 6, 7, and 12 by research associates (six trained nutritional scientists) [29]. Each component of every meal was weighed with digital kitchen scales (Soehnle 67,080 Page Profi, accuracy 1 g). If weighing of leftovers was not possible (e.g., mixing of components on the plate), quantities were estimated through household measures. All energy-containing drinks were documented. Snacks consumed during the night were recorded by nurses. Energy and protein intake were calculated with EbisPro 2016 (Willstätt-Legelshurst, Germany, German Nutrient Data Base Version 3.02).

Body weight (BW), handgrip strength and quality of life (QoL) (secondary outcomes) were assessed in weeks 1, 6, and 12.

*BW* was measured by nursing staff in residents wearing regular indoor clothing using available chair or lift scales.

*Handgrip strength* was measured with a Martin-Vigorimeter (Tuttlingen, Germany) using the medium-sized ball to the nearest of 0.2 kPA according to the manufacturers' standard operating procedure in a sitting position, as far as possible. Difficulties regarding functional and/or cognitive impairment were documented. Bedridden patients were supported by research associates. After careful instruction, residents performed two trials with both hands alternately, starting with the dominant hand, with maximum vigour [30]. The maximum value of the dominant hand was used for analyses.

*QoL* was evaluated using three subscales (care relationship, 7 items; positive affect, 6 items; negative affect, 3 items) and two additional items (does not want to eat, enjoys meals) of the Quality of Life in Dementia (QUALIDEM) questionnaire [31], which were perceived relevant regarding the intervention. Items were subjectively rated by nursing staff with four response options: never, rarely, sometimes, and frequently (Item scores 0–3). For some items, the answer option “not applicable” could be used as ratings may not be possible for people with severe dementia. Subscale scores were calculated separately by adding up item scores and then transformed to values between 0 and 100 for each subscale [32].

As specific items cannot be assessed in residents with *very* severe dementia [32, 33], a reduced number of items was applied in participants with “very severe cognitive decline” (Global Deterioration Scale [34] (GDS); see Ettema et al. [31]). GDS was evaluated by research associates.

### Statistics

Samples size was calculated for energy intake as the primary outcome (power 0.8, α = 0.05, two-sided, GPower, Düsseldorf). Previous studies indicated an average baseline intake of about 1300 ± 300 kcal [8, 35]. An increase of 200 ± 450 kcal was expected. Accordingly, 44 participants were needed to show significant effects. Assuming a dropout rate of 25% [8, 35], 55 residents were needed at entry.

Statistical analysis was performed using IBM SPSS Statistics for Windows, version 26 (IBM Corp., Armonk, NY). Only participants who completed the study with interruption of the intervention < 7 days and with complete outcome data were considered in the respective analyses.

Baseline characteristics are given as median and interquartile range (IQR) or mean and standard deviation (SD) for continuous variables and *n* (%) for categorical variables. Normal distribution was checked using normal *Q*–*Q* plot. Chi-squared test, *t*-test or Mann–Whitney-*U*-test were used to compare baseline characteristics between residents receiving regular and texture-modified meals.

Effects of the intervention on energy and protein intake, BW, handgrip strength and QoL were evaluated by analysis of variance (ANOVA) with repeated measures adjusted for multiple comparisons using Bonferroni correction (Model 1 (M1)). In case of significant results, post hoc tests were applied. An analysis of covariance (ANCOVA) with repeated measures was conducted by including supplement level (0–4) as grouping factor and Barthel-Index as covariate (M2). Subgroup analyses were conducted in residents receiving regular and reshaped texture-modified meals.

A *p*-value < 0.05 was considered statistically significant.

## Results

### Participants’ characteristics

We screened 306 residents, 55 were enrolled, and 50 completed the study (Supplement Fig. S2). Four participants died before the start of the intervention and one was in a hospital for > 7 days and not present during data collection of the primary outcome. Mean age of the participants was 84 years, 74% were female, 26% malnourished and 74% at risk (Table 1). Two residents received ONS, three received maltodextrin or enriched soups, and three a combination of both. A swallowing disorder was diagnosed by a physician in 12% of the residents and 32% received texture-modified meals. Participants consuming texture-modified meals had lower BMI, were more often malnourished, in need of eating assistance, and had more often functional and cognitive impairments compared to those receiving regular meals.

**Table 1:** Participants’ characteristics at baseline in the total sample and stratified by meal texture

	Total *n* = 50	Regular meals *n* = 34	Texture-modified meals *n* = 16	*p* value
Age [years], mean (± SD)	83.9 (± 7.9)	84.9 (± 8.0)	82.0 (± 7.5)	0.24^b^
Female gender, *n* (%)	37 (74.0)	26 (76.5)	11 (68.8)	0.56^a^
No medications per day, median (IQR)	6.0 (4–9)	7.0 (4–10)	5.0 (5–7)	0.07^c^
No chronic diseases, median (IQR)	4.0 (3–6)	4.0 (3–6)	5.0 (4–7)	0.15^c^
Barthel-Index [points], median (IQR)	40.0 (5–70)	55.0 (35–75)	5.0 (0–18)	< 0.001^c^
Clinical Frailty Scale [points], mean (± SD)	6.8 (± 0.9)	6.5 (± 0.9)	7.4 (± 0.5)	< 0.001^b^
Dementia, *n* (%)				
Severe	27 (54.0)	13 (38.2)	14 (87.5)	0.001^c^
Mild	15 (30.0)	13 (38.2)	2 (12.5)	
No	8 (16.0)	8 (23.5)	0 (0.0)	
Mobility, *n* (%)				
Bed/chair bound	18 (36.0)	6 (17.6)	12 (75.0)	< 0.001^c^
Able to get out of bed/chair	19 (38.0)	15 (44.1)	4 (25.0)	
Goes out	13 (26.0)	13 (38.2)	0 (0.0)	
Eating assistance, *n* (%)				
Partial or full	18 (36.0)	4 (11.8)	14 (87.5)	< 0.001^a^
None or guiding	32 (64.0)	30 (88.2)	2 (12.5)	
BMI [kg/m^2^], median (IQR)	22.6 (20–26)	23.3 (22–28)	20.3 (19–23)	0.03^c^
MNA-SF, *n* (%)				
Malnourished	13 (26.0)	4 (11.8)	9 (56.3)	0.001^a^
Risk of malnutrition	37 (74.0)	30 (88.2)	7 (43.8)	

*BMI* body mass index; *IQR* interquartile range; *MNA-SF* mini nutritional assessment-short form (malnourished 0–7 points, risk of malnutrition 8–11 points); *No* number; *SD* standard deviation

^a^Chi-Squared test

^b^*t*-test

^c^Mann-Whitney *U* test

### Supplementation

In phase 2, 258 ± 167 kcal and 23 ± 15 g protein were offered additionally per day. Ten (20%) participants received no supplementation. Four (8%) residents received supplementation level 1, 14 (28%) level 2, 10 (20%) level 3 and 12 (24%) level 4. Supplementation in the group with reshaped texture-modified meals was 287 ± 191 kcal and 26 ± 17 g protein and in the group with regular meals 244 ± 155 kcal (*p* = 0.09) and 22 ± 14 g protein (*p* = 0.11).

### Adverse events

In phase 1, seven (14%) residents had unspecific gastrointestinal complaints. During phase 2, six (12%) participants had gastrointestinal complaints that affected the intervention: supplementation was terminated in two, and interrupted for 1 week and then slowly resumed in four residents. Three (6%) residents were in the hospital for a maximum of two nights during phase 1 and five (10%) during phase 2, unrelated to the intervention.

### Dietary intake

**Fig. 2:**
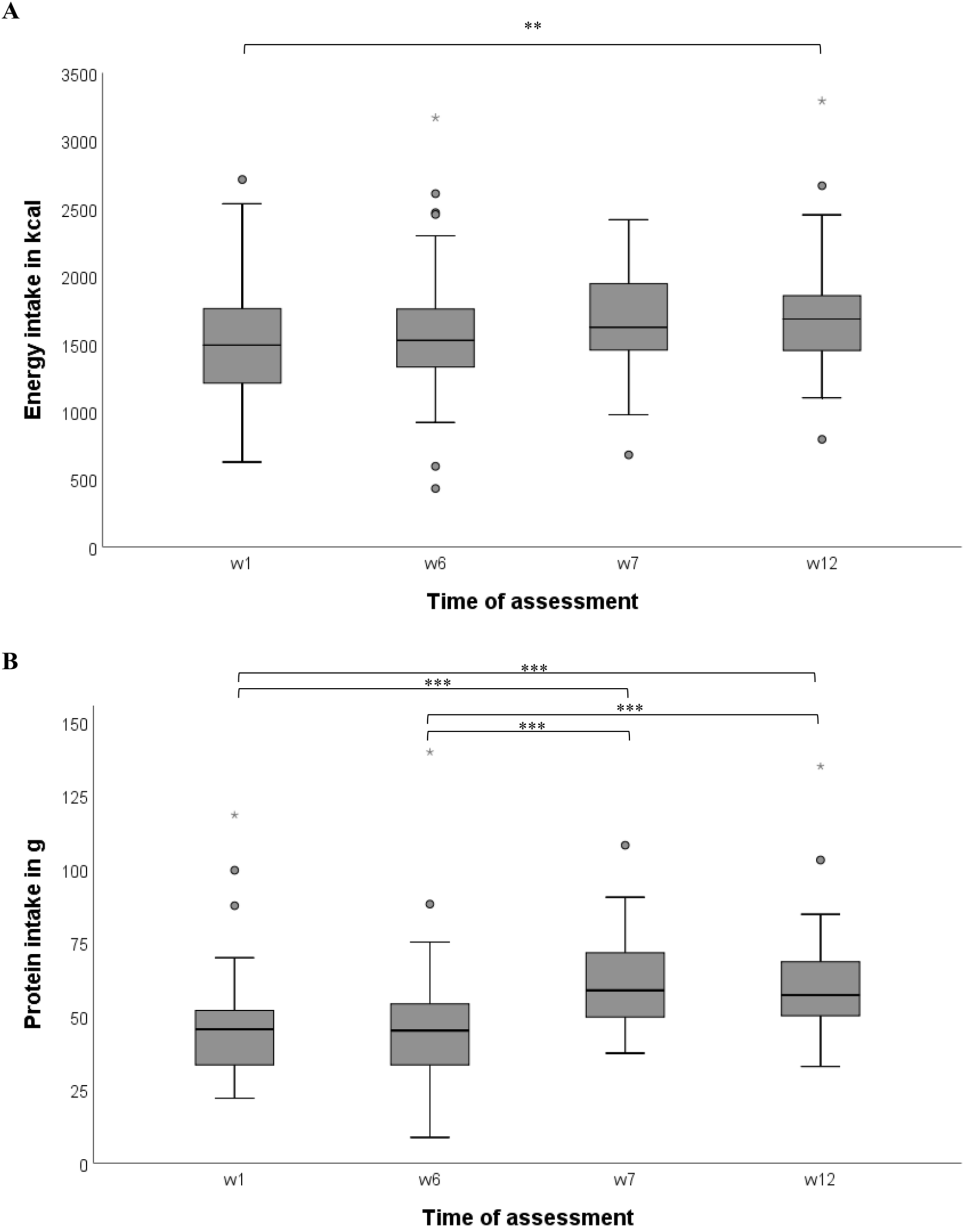
Boxplots of energy (**A**) and protein (**B**) intake (mean of 3 days) in weeks 1, 6, 7 and 12 *n* = 50; ANOVA with repeated measures adjusted for multiple comparisons: Bonferroni. Boxplots displaying (from outside to inside) extreme values (*), outliers (°), largest and smallest values within 1.5 times interquartile range above 75th and below 25th percentile, 75th, 25th (Interquartile range, grey box) and 50th (Median, horizontal line) percentile; **p < 0.01; ***p < 0.001. *w* week

Mean daily energy intake did not differ within the phases, but was higher in w12 compared to w1 (+ 207.2 kcal (95% CI 46.8–367.5 kcal), *p* = 0.005) (Table 2, Fig. 2A). Mean daily intake from food supplements was 162.9 (± 127.5) kcal in w7 and 137 (± 124.6) kcal in w12 (Table 2).

**Table 2:** Daily energy and protein intake (mean of 3 days) in weeks 1, 6, 7 and 12 in the total sample (total, from usual foods and supplements) and stratified by meal texture

Intake per day	*n*	Mean (± standard deviation)				M1 *p* value^a^	M2 *p* value^b^		
		w1	w6	w7	w12	Time	Time	SL	Time*SL
Energy									
Total [kcal]	50	1505.3 (± 440.4)	1567.5 (± 491.7)	1644.9 (± 346.1)	1712.5 (± 429.7)	0.001	0.07	0.001	0.001
From usual foods [kcal]^c^	50			1482.0 (± 381.8)	1575.5 (± 456.5)	0.067	0.141	0.004	0.275
From supplements [kcal]^c^	50			162.9 (± 127.5)	137.0 (± 124.6)	0.052	0.324	< 0.001	0.322
Total [kcal/kg bw]	50	24.5 (± 6.9)	25.5 (± 7.3)	27.0 (± 6.0)	28.1 (± 7.7)	< 0.001	0.024	0.214	< 0.001
RTMM [kcal]	16	1425.9 (± 455.4)	1484.2 (± 632.0)	1548.7 (± 425.8)	1798.8 (± 445.4)	0.009	0.13	0.042	0.22
Regular meals [kcal]	34	1542.7 (± 435.0)	1606.7 (± 415.3)	1690.1 (± 298.1)	1673.3 (± 423.2)	0.037	0.51	0.048	0.002
Protein									
Total [g]	50	46.7 (± 18.5)	46.9 (± 20.0)	61.3 (± 15.0)	60.6 (± 17.5)	< 0.001	< 0.001	0.017	< 0.001
From usual foods [g]^c^	50			46.5 (± 16.6)	48.2 (± 19.8)	0.347	0.948	0.002	0.348
From supplements [g]^c^	50			14.8 (± 12.0)	12.4 (± 11.5)	0.052	0.274	< 0.001	0.403
Total [g/kg BW]	50	0.75 (± 0.22)	0.75 (± 0.24)	1.01 (± 0.23)	0.99 (± 0.28)	< 0.001	< 0.001	0.37	< 0.001
RTMM [g]	16	41.0 (± 11.1)	42.1 (± 18.0)	57.4 (± 13.8)	60.3 (± 13.5)	< 0.001	0.08	0.16	0.048
Regular meals [g]	34	49.3 (± 20.6)	49.1 (± 20.6)	63.1 (± 15.4)	60.7 (± 19.2)	< 0.001	0.005	0.07	< 0.001

*BW* body weight; *SL* supplementation level; *M* model; *RTMM* reshaped-texture modified meals, *w* week

^a^M1: ANOVA with repeated measures, adjusted for multiple comparisons: Bonferroni

^b^M2: ANCOVA with repeated measures, adjusted for multiple comparisons: Bonferroni with SL as grouping factor and Barthel-index as covariate, SL: displays if variables were different in different supplementation levels, time*SL: indicates the interaction between time and supplementation level

^c^ANOVA and ANCOVA without repeated measures time: indicates if variables changed during the study

After including supplementation level and Barthel-Index in the analysis, we identified a difference in energy intake between the five levels and an interaction between supplementation level and time, with the highest increase in energy intake in level 4 (M2, Supplement Fig. S3, A). This interaction was also present in the subgroup of residents receiving regular meals (*p* = 0.002), but not in the group with reshaped texture-modified meals (*p* = 0.22) (Table 2). In phase 1, mean energy intake of residents with texture-modified meals was lower than intake of residents with regular meals, but in w12, this relation reversed (Table 2).

Mean daily protein intake did not differ within both phases, but was higher in w7 and w12 compared to w1 and w6 (+ 13.9 g (95%CI 6.9–21.0 g), *p* < 0.001, w12 vs w1) (Table 2, Fig. 2B). Mean daily intake from food supplements was 14.8 (± 12.0) g protein in w7 and 12.4 (± 11.5) g protein in w12 (Table 2).

In Model 2, a difference in protein intake between the supplementation levels and an interaction between levels and time was present, with a higher increase in protein intake in the higher levels (Supplement Fig. S3, B). Subgroup analyses revealed an increase in protein intake over time (M1) and interactions between supplementation level and time (M2) for the regular meal and the texture-modified meals group (Table 2).

### Body weight, handgrip strength and quality of life

Mean baseline body weight did not change throughout the study (M1, Table 3). BW clearly differed between supplementation levels in the total group (M2, Supplement Figure S4) and the subgroup receiving regular meals (M2, Supplement Table S2). Additionally, BW differed between levels over time (M2, Table 3).

**Table 3:** Body weight, handgrip strength and quality of life in weeks 1, 6 and 12

Variable	*n*	Mean (± standard deviation)			M1 *p* value^a^	M2 *p* value^b^		
		w1	w6	w12	Time	Time	SL	Time*SL
Body weight [kg]	50	62.3 (± 13.3)	62.3 (± 13.5)	62.7 (± 13.9)	0.33	0.90	0.045	0.013
Handgrip strength [kPa]	31	37.2 (± 14.6)	37.4 (± 18.3)	38.4 (± 17.4)	0.79	0.39	0.60	0.55
QUALIDEM								
Care relationship	49	76.2 (± 24.4)	73.4 (± 25.0)	82.0 (± 23.1)	0.004	0.010	0.34	0.26
Positive affect	49	75.3 (± 22.0)	71.8 (± 22.0)	72.3 (± 23.9)	0.52	0.16	0.68	0.58
Negative affect	49	75.4 (± 24.5)	75.3 (± 26.7)	76.6 (± 26.7)	0.91	0.17	0.81	0.044
Does not want to eat	48	61.1 (± 42.0)	64.6 (± 40.9)	59.7 (± 37.0)	0.67	0.29	0.11	0.63
Enjoys meals	42	87.3 (± 25.5)	70.6 (± 34.7)	66.7 (± 36.8)	0.001	0.004	0.22	0.95

QUALIDEM: Number of items subjectively rated by nurses per subscale: Care relationship (7 items), positive affect (6 items), negative affect (3 items), does not want to eat (1 item), enjoys meals (1 item)

*SL* supplementation level; *M* model; *QUALIDEM* quality of life in dementia (scale 0–100 points); *w* week

^a^M1: ANOVA with repeated measures, adjusted for multiple comparisons: Bonferroni

^b^M2: ANCOVA with repeated measures, adjusted for multiple comparisons: Bonferroni, with SL as grouping factor and Barthel-index as covariate time: indicates if variables changed during the study, SL: displays if variables were different in different supplementation levels, time*SL: indicates the interaction between time and supplementation level

Measurement of handgrip strength was not possible in 38% of the participants due to severe dementia (*n* = 8), functional limitations (*n* = 5), or a combination of both (*n* = 6). In residents with available data, mean handgrip strength did not change throughout the study (Table 3).

In ten residents, the reduced QUALIDEM version for persons with very severe dementia was used. QoL was not assessed in one resident due to a lack of verbal and non-verbal communication (very severe Parkinson’s disease). Items “does not want to eat” and “enjoys meals” were “not applicable” for two and eight residents, respectively. The mean score of the subscale “care relationship” increased from w6 to w12 (+ 8.6 (95% CI 2.8–14.5) points, *p* = 0.002) (M1, Supplement Fig. S5, A). The effect was significant in residents receiving reshaped texture-modified meals, but not in those with regular meals (M1, Supplement Table S2). The subscales “positive affect”, “negative affect” and the item “does not want to eat” did not change throughout the study (Table 3). For the subscale “negative affect” an interaction between supplementation level and time was observed (M2, Table 3), but without clear direction (M2, Supplement Fig. S5, B). The mean score for the item “enjoys meals” decreased throughout the study (M1, Table 3). Post hoc tests showed that the decrease occurred in phase 1, but not in phase 2 (w1 vs w6, −16.7 (95% CI −30.6–(−2.7)), *p* = 0.015; w1 vs w12, −20.6 (95% CI −33.0–(−8.3)), *p* < 0.001; w6 vs w12, −4.0 (95% CI −19.6–11.6), *p* = 1.00). This decrease was observed in both meal-texture subgroups (M1, Supplement Table S2).

## Discussion

In the present study, the individualised nutritional intervention consisting of three supplement modules and reshaped texture-modified meals improved energy and protein intake of nursing home residents with (risk of) malnutrition. During the intervention phase, participants averagely met the reference value for protein intake (1 g/kg BW/d), and averagely almost reached their estimated energy requirements (28 ± 8 vs 30 kcal/kg BW/d) [15].

We explicitly included individuals with chewing and/or swallowing difficulties and dementia to support adequate nutrition for residents with these risk factors of malnutrition [12]. Consequently, in our sample, cognitive status was lower compared to other studies in the nursing home setting [18, 35], but gender, age and BMI were similar [18, 35, 36]. Besides the higher number of residents with dementia, also compared to the usual German nursing home population [37–39], a large proportion of bedridden (36%) and functionally impaired individuals, especially in the group with texture-modified meals, was striking in the present population.

Energy and protein intake increased by 207 kcal and 14 g when comparing the last to the first study week. With our individualised approach, we aimed to adequately address calculated energy and/or protein deficiencies rather than increasing intakes as much as possible. We tried to meet the calculated deficiencies and only increased the supplementation above the calculated deficiency in case of low BMI and desired weight gain, decided in individual case discussions [20]. Noteworthy, protein and energy requirements were estimated and not directly measured, which entails inaccuracies [40]. Plausibly, participants in higher supplementation levels had a higher increase in energy and protein intake (Supplement Fig. S3); furthermore, effects were more pronounced in residents with texture-modified meals (Table 2), which may be explained by slightly greater deficiencies [20] and resulting higher supplementation as well as by greater mealtime assistance, allowing nurses to focus on the consumption of the intervention products.

In the present analysis, energy and protein intake from usual food sources as well as from supplementation did not change within the intervention phase (Table 2). In a secondary data analysis, limited to residents receiving a supplementation (*n* = 40), we examined the effects of the intervention on protein intake in more detail and identified that the supplementation did not affect protein intake amount and sources from regular food components during the intervention phase compared to the usual care phase [23].

Compliance with the intervention was acceptable, however, consumption varied between modules. Median intake of the offered amount was lowest for the savoury protein cream (44%), higher for the sweet protein cream (61%) and highest for the protein-energy drink (76%). (Median intake of 6 days based on weighed food records in the first and last week of the intervention phase) [20], and was thus comparable to compliance with commercial ONS [41]. About half of the staff reported being able to integrate the intervention products into their routine. Acceptability by residents and staff was higher for the protein-energy drink than for the protein creams [20], probably explained by greater familiarity with drinks like ONS.

Besides dietary intake, we assessed effects on other outcomes. QoL in one subscale increased, but BW and handgrip strength did not change during the study. Lacking body weight change was not surprising as only 40% of the participants had the objective to gain weight and we individualised supplementations accordingly [20]. Furthermore, other studies in nursing homes with longer durations also did not observe significant effects on BW [18, 42] and prevention of weight loss may already be seen as a success when considering the natural progressive decline in muscle mass, strength and function associated with ageing [43].

Assessment of handgrip strength was not possible in more than two-third of the present population, even though we preferred the Martin-Vigorimeter to a dynamometer. Measurements with a Vigorimeter are assumed to be easier in participants with cognitive and/or functional limitations [30] and moderate to strong correlations with dynamometer measurements have been reported [44]. Missing effects in our and other nutritional intervention studies may be explained by difficulties in measurement of handgrip strength with the dynamometer [17, 18, 36, 45] as well as with the Martin-Vigorimeter [35].

Quality of life is a very important outcome, but also difficult to assess in this particular population with more than 50% severe and more than 80% at least mild dementia. Therefore, it was necessary to rely on proxy-ratings. We used the QUALIDEM, which was considered feasible and showed moderate to high internal consistency [46]. In addition, the development of a user guide improved inter-rater reliability [47]. However, the authors state that some subscales need revision [32]. Effects on QoL need therefore to be interpreted with caution. QoL in the "care relationship" subscale improved during our intervention phase by 9 (95%CI 3–15) points. The effect may be explained by increased attention and awareness of nurses about individual nutritional problems and needs in addition to the increased dietary intake. This hypothesis is supported by more pronounced effects among residents receiving texture-modified meals, who required more mealtime assistance in comparison to those receiving regular meals (Table 1). The item score “enjoys meals” deteriorated by 17 points in the usual care phase. This negative association suggests that other factors, namely “residents challenging behaviour, nurses’ burnout, and satisfaction with life” [48], may influence proxy-ratings of QoL, which shows the limitations and restricted reliability of these assessments.

A major strength of the present intervention concept is that we addressed the enjoyment of meals, in particular regarding the reshaped texture-modified meals, but also regarding our food supplements. The protein creams were produced freshly every day, and the protein-energy drink was developed based on the sensory preferences of older adults [49]. It was hygienically produced by food technologists in small scale for the present study. Furthermore, the intervention concept was individualised and easy to implement due to its modular structure. However, the intervention concept could be optimised for future studies, for example by developing an easy-to-use instrument to screen for deficiencies or by additionally offering supplements at dinner. Moreover, a reduced number of supplement levels might be sufficient. It might furthermore be necessary to offer protein products, without additional energy supplementation, e.g., for older adults with sarcopenic obesity.

The present study has several limitations that need to be addressed. We did not randomize participants into an intervention and a control group due to practical constraints regarding meal provision, the great heterogeneity of the target population and ethical concerns. Instead, we used a sequential design in which every participant served as its own control and which allowed every resident to receive an intervention. The duration of each study phase was limited to 6 weeks as we wanted to reduce the risk of a decline in residents' health status. Additionally, in a proof-of-concept study with the same duration, we were able to observe positive effects on energy and protein intake as well as body weight [8]. When considering a controlled parallel-group study design and focusing on functional outcomes, a longer study duration would be desirable. Furthermore, blinding of nursing home personnel was not possible as they provided the nutritional intervention, which could not be replaced by placebo products. Blinding of research associates was not possible as they conducted the 3-day weighed food records. Additionally, it is important to mention that regarding QoL we relied on information from nursing staff, as assessments with residents were not possible due to the high rate of dementia. Nutritional deficiencies were assessed at baseline and might have changed during the usual care phase. Due to staff constraints, deficiencies could not be reassessed before the start of the intervention. However, mean dietary intake did not change during the usual care phase implying also an unchanged deficiency situation. Finally, it is not possible to distinguish between the effects of the supplementation and the reshaped texture-modified meals, as we combined the intervention modules according to individual needs.

In conclusion, the present study is one of the first examining the effects of an individualised modular nutritional intervention in nursing home residents with malnutrition or risk of malnutrition. These first insights show that the intervention concept with individualised supplementation and reshaped texture-modified meals can improve energy and protein intake and QoL with regard to care relationship. The effects may be explained by the nutritional supplementation and by potentially increased support and awareness from nurses. Our modular concept could be further developed for other nursing homes. Future studies with larger samples, longer duration and, most important, controlled design are needed to confirm the results and examine superiority of individualised compared to established standardised interventions (e.g., ONS).

## Supplementary Information

Below is the link to the electronic supplementary material.

Supplementary file1 (PDF 300 KB)

## Data Availability

The datasets generated during and/or analysed during the current study are available from the corresponding author on reasonable request. The data are not publicly available due to residents or legal representatives not giving full consent.
