# Supplementary material for: Effects of an individualised nutritional intervention to tackle malnutrition in nursing homes: a pre-post study
Source: Eur Geriatr Med. 2021 Dec 1;13(3):741–52. doi: 10.1007/s41999-021-00597-y (PMC9151515; doi:10.1007/s41999-021-00597-y)
Supplement: Supplementary file 1 — Supplementary file1 (PDF 300 KB) [file 41999_2021_597_MOESM1_ESM.pdf]

Supplementary Material

Seemer J et al. (2021) Effects of an individualised nutritional intervention to tackle malnutrition in nursing homes: a pre-post study

Structure of the NH's:

- Number of NH's: 2
- Number of nursing wards: 9
- Number of buildings: 4
- Number of beds
  - Per ward: Median 29 (Range 27-59)
  - Total: 312 (including short-term care)
- Staff: Nursing home management (n=2), quality assurance (n=2), dietitian (n=1), nursing staff management (n=4), nursing ward management (n=9), registered nurses (n=73), nursing aides (n=76), social care workers (n=18), housekeeping staff (n=11)

Usual nutritional care of the NH's:

- NH's received meals from one central kitchen
- 3 main meals (breakfast, lunch, and dinner) and additional snacks
- Regular meals:
  - Breakfast: Based on bread and pastries with butter, cold cuts, cheese, jam; Optional: Yoghurt
  - Lunch (main meal): Tray-based catering, 8-week meal plan (3 menu lines, incl. 1 vegetarian)
  - Dinner: Based on bread with butter, cold cuts, cheese and daily specials (e.g., raw vegetable salads, pickled salads or fish); Optional: Yoghurt and/or soup
- Texture modified meals:
  - Breakfast: Nursing staff prepared porridge on the wards based on milk and instant cereal or fruit flakes; Optional: Yoghurt
  - Lunch: Tray-based catering, 8-week meal plan (2 menu lines), meal components were pureed with added water or milk and texturized with potato powder, Spoons were used to shape and place meal components on the plate
  - Dinner: Kitchen produced porridge based on milk and either oats, maize, semolina, millet or rice, and served it with fruit compote; Optional: Yoghurt and/or soup
- Afternoon snack: varying baked goods (e.g., plane cake, milk snack, apple pie)
- Other snacks: Fruit and yoghurt; Food brought by family and friends or bought by themselves
- Beverages:
  - Water and juice were available at all times
  - Breakfast and afternoon snack: Coffee or tea were provided
  - Dinner: Tea was provided
- Supplements: If perceived necessary according to nurses' or physicians' subjective assessment, residents were offered
  - Energy-enriched soup instead of a usual soup and/ or
  - Oral nutritional supplements (for a short period <1 week financed by NH, for longer periods >1 week financed by resident/ legal representative) and/ or
  - Maltodextrin stirred into meals by nurses

Fig S1 Information box: Structure and usual nutritional care of the participating nursing homes (NH)

For further information see: [20] Seemer J, et al. An Individualised Nutritional Intervention Concept for Nursing Home Residents with or at Risk of Malnutrition: An enable Study. Geriatrics. 2021;6(1):12. https://doi.org/10.3390/geriatrics6010002. [23] Seemer J, et al. Usual Protein Intake Amount and Sources of Nursing Home Residents with (Risk of) Malnutrition and Effects of an Individualized Nutritional Intervention: An enable Study. Nutrients. 2021;13(7). https://doi.org/10.3390/nu13072168.

Tab S1 Energy and protein deficiency, supplement modules and their energy and protein content in 5 supplementation levels [20].

Level	Deficiency		Supplementation		Offered supplement modules
	Energy [kcal]	Protein [g]	Energy [kcal]	Protein [g]	
**0**	<0	<0	0	0	-
**1**	0-150	0-10	+125	+10	Sweet protein cream
**2**	151-250	11-20	+220-250	+20-22	(Sweet and savoury protein cream) or protein-energy drink
**3**	251-350	21-30	+345	+32	Sweet protein cream and protein-energy drink
**4**	>350	>30	+470	+42	Sweet and savoury protein cream and protein-energy drink

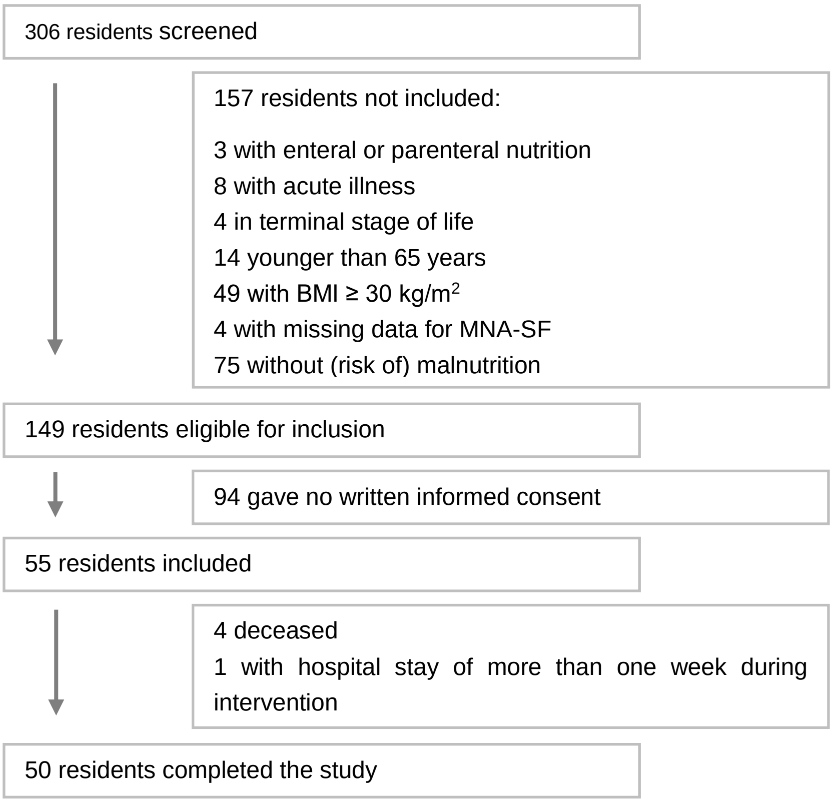

Fig S2 Flow chart of study process

BMI=Body Mass Index, MNA-SF=Mini Nutritional Assessment-Short Form

A

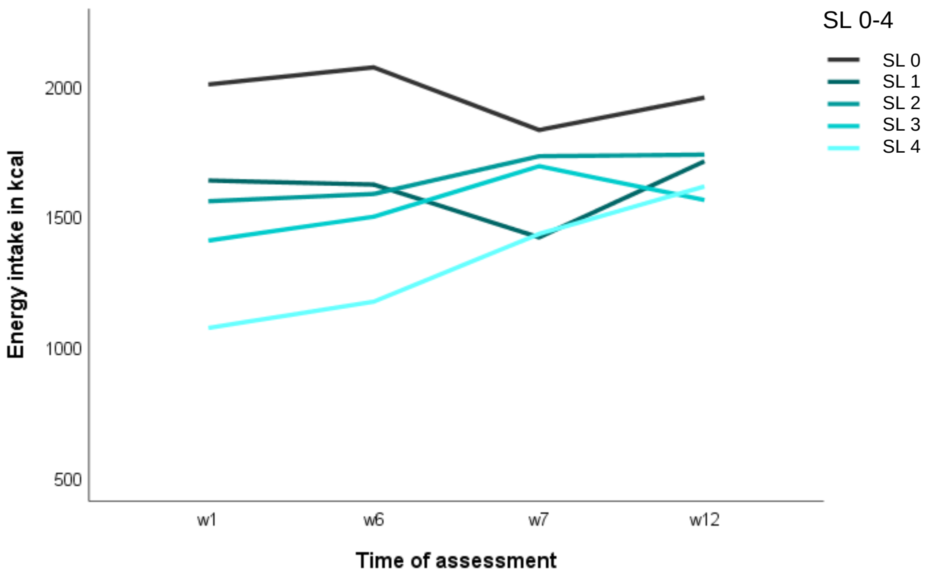

B

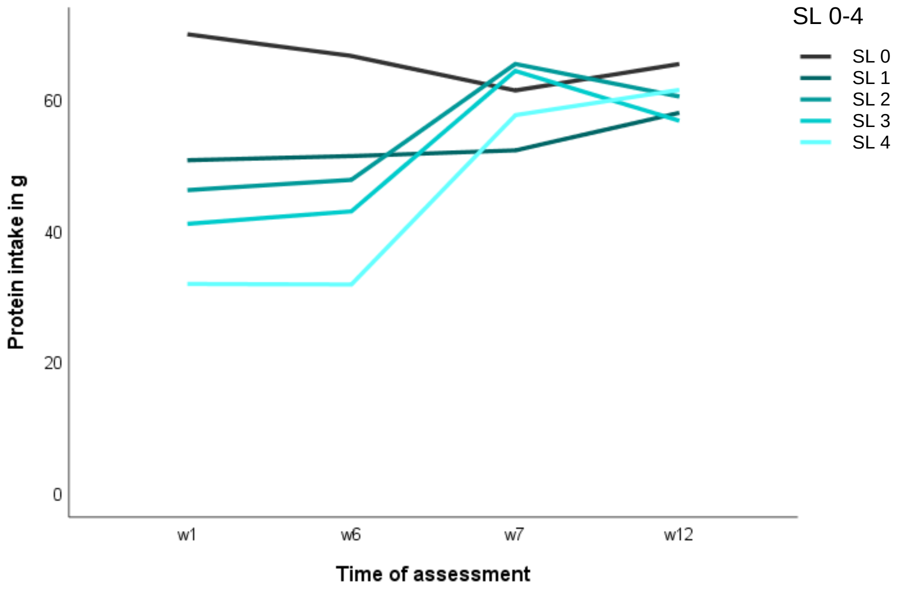

Fig S3 Estimated marginal means of energy (A) and protein (B) intake (mean of 3 days) in weeks 1, 6, 7 and 12 stratified by supplementation level

n=50; ANCOVA with repeated measures, adjusted for multiple comparisons: Bonferroni with SL as grouping factor and Barthel-index as covariate. Number of residents per supplementation level: SL0: n=10, SL1: n=4, SL2: n=14, SL3: n=10, SL4: n=12

SL=supplementation level, w=week

Tab S2 Body weight, handgrip strength and quality of life in weeks 1, 6 and 12 stratified by meal texture

				Mean (± standard deviation)		M1 p-value[a]	M2 p-value[b]		
**Variable**	**Subgroup**	n	w1	w6	w12	time	time	SL	time * SL
**Body weight** [kg]	RTMM	16	56.7 (±8.6)	56.7 (±8.6)	57.1 (±8.9)	0.72	0.20	0.42	0.28
	Reg meals	34	65.0 (±14.4)	64.9 (±14.7)	65.4 (±15.1)	0.44	0.43	**0.045**	0.10
**Handgrip strength** [kPa]	RTMM	4	37.5 (±13.3)	31.0 (±16.1)	33.5 (±13.8)	0.52	[c]		
	Reg meals	27	37.2 (±16.3)	38.4 (±18.7)	39.1 (±18.0)	0.59	0.46	0.72	0.70
**QUALIDEM** **Care relationship**	RTMM	15	76.5 (±25.7)	76.6 (±20.0)	91.0 (±12.7)	**0.017**	0.77	0.10	0.46
	Reg meals	34	76.1 (±24.2)	72.0 (±27.1)	77.9 (±25.6)	0.09	0.48	0.60	0.85
**Positive affect**	RTMM	15	67.0 (±18.6)	67.0 (±19.3)	65.3 (±19.6)	0.94	0.88	0.79	0.88
	Reg meals	34	78.9 (±22.6)	73.9 (±23.0)	75.3 (±25.3)	0.46	0.11	0.49	0.76
**Negative affect**	RTMM	15	72.2 (±26.0)	56.2 (±26.3)	71.9 (±31.9)	0.55	0.24	0.95	0.20
	Reg meals	34	76.8 (±24.1)	79.7 (±26.0)	78.8 (±24.4)	0.76	0.09	0.70	0.24
**Does not want to eat**	RTMM	14	45.3 (±44.5)	47.6 (±38.6)	52.4 (±31.3)	0.83	0.10	0.33	0.42
	Reg meals	34	67.6 (±39.8)	71.6 (±40.3)	62.7 (±39.1)	0.36	0.17	0.28	0.47
**Enjoys meals**	RTMM	11	90.9 (±21.6)	63.7 (±40.7)	57.6 (±33.6)	**0.040**	0.50	0.81	0.72
	Reg meals	31	86.2 (±26.9)	73.1 (±32.7)	69.9 (±37.9)	**0.026**	0.06	0.28	0.96

[a]M1: ANOVA with repeated measures, adjusted for multiple comparisons: Bonferroni [b]M2: ANCOVA with repeated measures, adjusted for multiple comparisons: Bonferroni with SL as grouping factor and Barthel-index as covariate [c]Statistical testing was not possible due to small number of participant

time: indicates if variables changed during the study, SL: displays if variables were different in different supplementation levels, time*SL: indicates the interaction between time and supplementation level

QUALIDEM: Number of items subjectively rated by nurses per subscale: Care relationship (7 items), Positive affect (6 items), Negative affect (3 items), Does not want to eat (1 item), Enjoys meals (1 item);

SL=supplementation level, M=Model, QUALIDEM=Quality of Life in Dementia (scale 0-100 points), Reg=regular, RTMM=reshaped texture modified meals, w=week

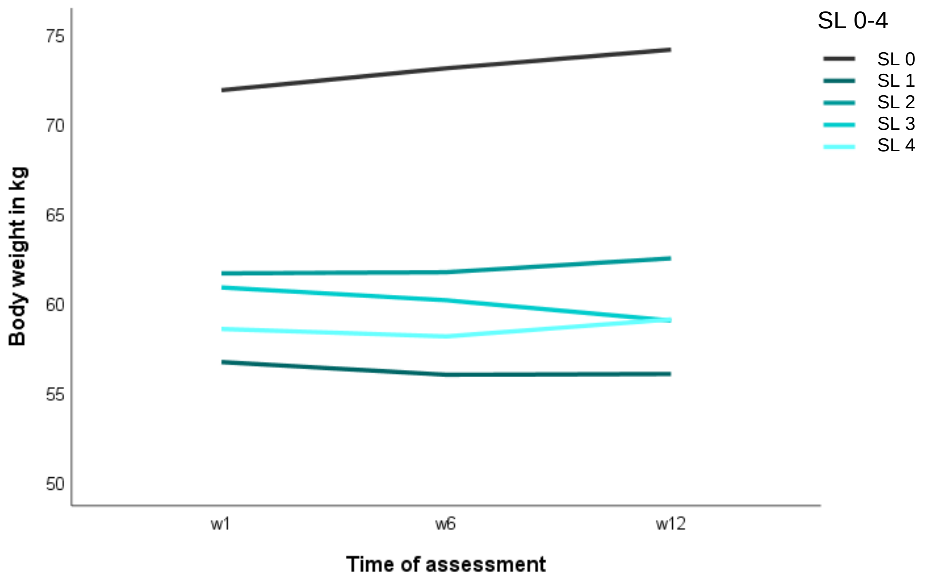

Fig S4 Estimated marginal means of body weight in weeks 1, 6 and 12 stratified by supplementation level

n=50, ANCOVA with repeated measures, adjusted for multiple comparisons: Bonferroni with SL as grouping factor and Barthel-index as covariate. Number of residents per supplementation level: SL0: n=10, SL1: n=4, SL2: n=14, SL3: n=10, SL4: n=12

SL=supplementation level, w=week

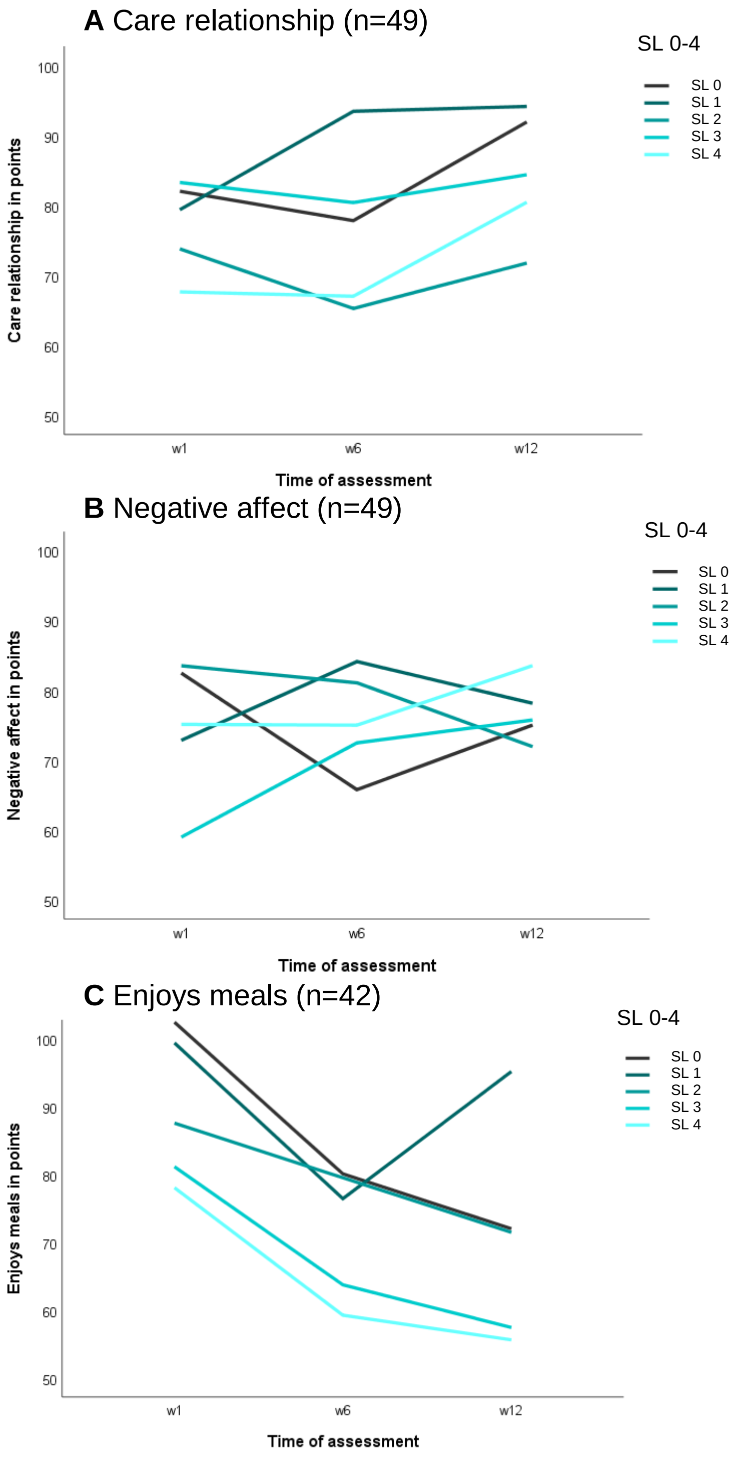

Fig S5 Estimated marginal means of quality of life (A-C) in weeks (w) 1, 6 and 12 stratified by supplementation level (SL)

ANCOVA with repeated measures, adjusted for multiple comparisons: Bonferroni, with SL as grouping factor and Barthel-index as covariate. Number of residents per supplementation level for A and B SL0: n=9, SL1: n=4, SL2: n=14, SL3: n=10, SL4: n=12; for C: SL0: n=7, SL1: n=4, SL2: n=11, SL3: n=9, SL4: n=11

SL=supplementation level, w=week
